# Supplementary material for: Assessment of Body Composition and Physical Performance of Young Soccer Players: Differences According to the Competitive Level
Source: Biology (Basel). 2022 May 27;11(6):823. doi: 10.3390/biology11060823 (PMC9219641; doi:10.3390/biology11060823)
Supplement: Supplementary file 1 [file biology-11-00823-s001.zip › biology-1676357-supplementary.pdf]

Supplementary materials

Table S1. Leave-one-out average-posterior-probabilities classification

		LOO Classified	
	True	Bologna	Russi
Number	Bologna	72	14
Average posterior prob.		0.896	0.729
Number	Russi	8	42
Average posterior prob.		0.76	0.862
Number	Total	80	56
Average posterior prob.		0.883	0.829
	Priors	0.5	0.5

Note: prob., probabilities; LOO, Leave-One-Out

Table S2. MANOVA and univariate ANOVA summaries

**MANOVA** (*n* =136)				
Statistic	value	df	$F_{(7, 128)}$	P
Wilks' lambda (W)	0.486	1	19.37	<0.001*
Pillai's trace (P)	0.514		19.37	<0.001*
Lawley-Hotelling trace (L)	1.059		19.37	<0.001*
Residual		134		
Total		135		

**ANOVA** (*n* =136)							
Variable	Model MS	Resid MS	Total MS	R^2	Adj. R^2	$F_{(1, 134)}$	P
Triceps SK	1.45	14.89	14.79	0.089	0.082	13.06	<0.001*
Biceps SK	0.88	6.64	6.6	0.117	0.111	17.78	<0.001*
Suprailiac SK	1.6	15.19	15.09	0.095	0.088	14.09	<0.001*
Medial Calf SK	1.45	13.91	13.82	0.095	0.088	14.01	<0.001*
CMJ test	691.70	3901.16	3877.39	0.151	0.144	23.76	<0.001*
Sprint 15m test	4.27	6.25	6.23	0.406	0.401	91.55	<0.001*
RSA 20+20m	12.16	28.71	28.59	0.298	0.292	56.78	<0.001*

Note: df, degree of fredom; MS, Mean Squared; Resid, Residual; n=number of observation; *, statistically significant

Table S3. Canonical LDA and Standardized function coefficients

Function	Canon correl.	Eigenvalue	Variance	LLR	$F_{(7, 128)}$	*P*
1	0.717	1.06	1	0.486	19.37	<0.001*
Standardized function coefficients						
Triceps SK	Biceps SK	Suprailiac SK	Med Calf SK	CMJ	Sprint 15m	RSA 20+20m
0.23	0.02	-0.5	0.04	-0.45	-2.39	1.26

Note: LDA, Linear Discriminant Analysis; Canon. correl., Canonical correlation; LLR, Likelihood Ratio; df, degree of freedom; *, Statistically significant
